# Informational support for women with endometriosis: a scoping review

**DOI:** 10.1186/s12905-025-03581-x

**Published:** 2025-02-03

**Authors:** Deniz Senyel, James H. Boyd, Melissa Graham

**Affiliations:** Department of Public Health, School of Psychology and Public Health, La Trobe University, Melbourne, Australia

**Keywords:** Information, Endometriosis, Women’s health, Information seeking behaviour

## Abstract

**Background:**

Ten per cent of women of reproductive age suffer from endometriosis, a painful and incurable disease that leaves women with severe implications for their health and overall well-being. Due to the absence of a cure and the limited effectiveness of available treatments, acquiring accurate information is paramount for women to successfully navigate both their daily lives and the complexities of the healthcare system. This scoping review aimed to map the current literature on women with endometriosis information needs, their information seeking behaviour, and the format and scope of current information resources available.

**Methods:**

The scoping review was conducted using the JBI methodology for scoping reviews and reported according to the PRISMA-ScR statement. The final search was conducted in August 2024, through the databases Medline, Cinahl, Embase, Scopus, and WebofScience. Studies on information resources on endometriosis and information seeking behaviour as well as information needs of women with endometriosis were eligible for inclusion.

**Results:**

The majority of the 25 included studies focused on information resources, specifically webpages and social media sites. While few studies analysed information seeking behaviour and information needs, the evidence shows women’s high interest in a broad spectrum of information topics. Across all studies, the internet was the most important access point for information.

**Conclusion:**

Addressing the absence of systematic analyses on the information seeking behaviour and needs of women with endometriosis is crucial for future research. This step is essential for the development of customised information resources that cater specifically to the diverse needs of women affected by endometriosis.

## Background

It is estimated that approximately 10 per cent of women^1^ of reproductive age suffer from endometriosis, a chronic and debilitating disease [1]. Endometriosis occurs when tissue similar to the lining of the uterus (endometrium) grows outside of the uterus, causing a spectrum of distressing symptoms [2]. These include but are not limited to dysmenorrhea, dyspareunia, chronic pain, infertility, and gastrointestinal issues [3]. Beyond its physical implications, endometriosis can also impact mental well-being and has been linked to mental illnesses such as depression and anxiety [4, 5]. Due to the multitude of symptoms, all aspects of a woman’s life can be affected. One consequence of the symptom burden is frequent absenteeism from work, often due to chronic pain [6, 7]. Additionally, women’s private life is often affected as they struggle to participate in social activities and are hindered by society’s stigma and misconceptions surrounding menstrual health and reproductive conditions [8–10]. This can create an environment where women feel hesitant to discuss their condition, leading to feelings of isolation and inaccessible social interactions. In the sphere of intimate relationships, the implications of endometriosis extend to fertility issues and a compromised sexual experience due to painful intercourse [11]. This can strain romantic relationships, causing further stress.

Endometriosis is currently incurable and effective treatment options are limited. The primary approaches typically involve surgical intervention for lesion removal, hormonal therapy, and the use of pain medication [3]. However, these measures often fail to provide lasting relief from symptoms. Therefore, a more holistic and multidisciplinary approach needs to be taken to address the complex and unique care needs of each woman and to find a treatment plan that restores quality of life [12, 13]. Due to the existing inadequacies in conventional treatment methods [14, 15] many women resort to various self-management strategies, which may include seeking support from allied healthcare services, exploring alternative medicine, and making dietary adjustments. Additionally, to alleviate pain women often turn to remedies such as the application of heat packs, sufficient rest, engaging in breathing exercises, and meditation [16].

There is a lack of understanding, awareness, and knowledge among healthcare providers and society which can have a detrimental effect on the care and well-being of women with endometriosis. Research shows that healthcare providers often lack sufficient knowledge about endometriosis [17, 18]. Further, women have reported dismissive attitudes from their healthcare providers, which has led to prolonged diagnostic times and inadequate care [19]. This can be caused by societal stigmatisation surrounding menstrual health, including a disregard for women’s pain [8]. Women with endometriosis may not only lack support from their healthcare providers but also from their social networks. Due to the lack of awareness in society and the existing stigma, women can be hindered from seeking help from friends, family or colleagues [20].

Together the lack of effective treatment options and the lack of support from healthcare providers and society results in women having to take care of their disease themselves. To be able to self-manage their disease, women need reliable and extensive information regarding endometriosis. This foundational knowledge can empower women to recognise and seek help for their symptoms [21]. An informed and empowered patient is vital to improve patient access to health services and better patient-provider communication [22]. Therefore, patient-led resources need to be developed to meet patients’ information needs, preferably aligned with their information seeking behaviour [23].

To our knowledge, there are currently no reviews addressing the area of informational support for women with endometriosis. This scoping review aims to map the current literature on women with endometriosis information needs, their information seeking behaviour, and the format and scope of current information resources.

## Methods

The scoping review was conducted in accordance with the JBI methodology for scoping reviews [24] and is reported according to the PRISMA-ScR statement [25]. A scoping review methodology was chosen as it can provide an overview of what has been researched so far and what research gaps remain. This is an appropriate first step to understanding the research area and to guide future research efforts.

### Review question

Based on our rationale, the following research questions were advanced:

- What are the information needs of women with endometriosis?
- What are the format and scope of information resources that have been described in the literature for women with endometriosis?
- Where do women with endometriosis seek and access information about endometriosis?

### Eligibility criteria

#### Participants

Studies were eligible for inclusion if they involved women with endometriosis. If studies included multiple patient groups a clear subgroup analysis for women with endometriosis needed to exist. Otherwise, these studies were excluded. Additionally, studies focusing on information resources were eligible if the target audience was women with endometriosis. No further age, socio-demographic variables, ethnicity or religion restrictions were made.

#### Concept

Information needs are a highly complex concept, influenced by a multitude of variables, both internal and external to the patient. These needs primarily emerge as a response to specific triggers or antecedents [26] and in the context of endometriosis, these triggers can include a diagnosis or suspicion of endometriosis, as well as the manifestation of related symptoms. Information needs can be defined as “a recognition that your knowledge is inadequate to satisfy a goal that you have, within the context/situation that you find yourself at a specific point in the time” [23]. This definition highlights that information needs are goal oriented and are highly influenced by the circumstances. Information needs are dynamic and can vary significantly from one individual to another. These needs are individualistic and subjective, reflecting the unique experiences, circumstances, and preferences of each person.

It should be noted that even though this definition was designed for the healthcare setting, it does not limit information needs to health information. As previously mentioned, endometriosis affects all parts of a woman’s life, thus the information needs included in this scoping review are not limited to health or the healthcare system.

Information seeking behaviour is the natural response to a patient’s information needs [27]. The way individuals seek information can vary widely, depending on the individual’s personal characteristics and specific circumstances. Information seeking behaviour can be broken down into two main components: the information dimension, which pertains to what information is being sought, and the method dimension, which involves how individuals go about seeking information and the information origins, such as personal and impersonal sources [28].

Based on these definitions the inclusion criteria were studies that:

- Analysed the information needs of women with endometriosis. Due to the inherently subjective nature of the topic proxy answers were not eligible [26].
- Described information seeking behaviour. These encompassed studies describing what types of information women with endometriosis seek and how and where the information is being sought.
- Reported information sources for women with endometriosis. Information sources could be personal or impersonal [28]. Personal sources included family, friends, and other personal contacts. Impersonal sources included every information source that is either non-human or a person without any personal relation to the woman. Further, non-human information sources could be digital or in print, with no restriction regarding the publication type. If the information included multiple topics, such as menstrual health in general, a separate analysis for the endometriosis content needed to be included.

#### Context

There were no limitations regarding the study context. Studies from any country were considered eligible for inclusion as long as the articles were written in English or German, the languages spoken by the research team. Articles written in languages other than English or German were excluded.

#### Types of sources

Both primary and secondary literature were eligible for inclusion. Secondary literature refers to any review summarising primary evidence from relevant studies. For primary literature both qualitative and quantitative studies were eligible. The eligible studies could be experimental or observational.

Abstracts without full-text available, expert opinions, commentaries, protocols, and case studies were excluded, as these did not provide sufficient information of relevance to this scoping review.

#### Search strategy

The search string was designed in Medline by the first author and reviewed by a senior librarian. Literature was sought in Medline, Cinahl, Embase, WebofScience, and Scopus. The first part of the search string consisted of the terms “endometriosis” and “endometrioma” to describe the disease. The equivalent MeSH term was also included. The second part described information needs, seeking behaviour, and resources. For this, the adjacent operator was used to combine the words information or knowledge with relevant keywords to create all possible combinations. This strategy delivers more results than using phrase searching. The example search in Table 1 shows the keywords used. This search was combined with all relevant MeSH terms. A few selected keywords were added that showed promising results during preliminary searches. The keyword function was used sparingly as words such as “information” or “knowledge” alone resulted in too many unrelated articles. The final search was conducted on the 5th of August 2024.

**Table 1:** Search strategy in medline

Set	Search Statement
1	((Information or knowledge) adj2 (finding or seek* or source* or hand-out or "hand-out" or patient or need* or behavio?r or distribution or search* or health or preference* or wish* or desire*)).mp
2	Consumer Health Information/
3	Information Dissemination/
4	Information Sources/
5	Information Seeking Behavior/
6	Social Media/
7	Health Communication/ or Mass Media/
8	Search Engine/
9	Patient Education as Topic/ or Patient Education Handout/
10	Internet/
11	Knowledge/
12	Education/
13	Health Education/
14	1 or 2 or 3 or 4 or 5 or 6 or 7 or 8 or 9 or 10 or 11 or 12 or 13
15	internet.mp
16	"social media".mp
17	"patient education".mp
18	"health information".mp
19	14 or 15 or 16 or 17 or 18
20	Endometriosis/
21	(endometriosis or endometrioma*).mp
22	20 or 21
23	19 and 22

#### Study selection

**Fig. 1:**
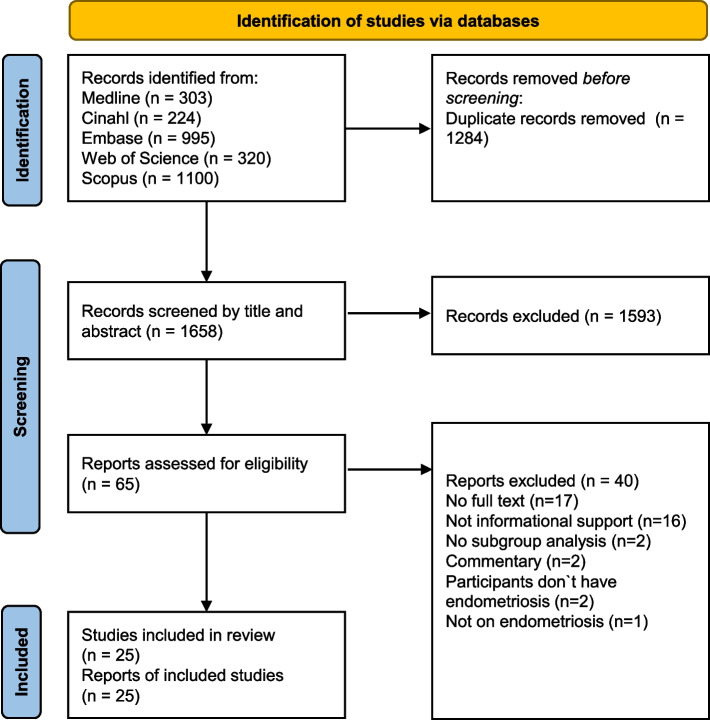
PRISMA flow chart of the scoping review process

The search strategy results were uploaded to Covidence for study selection. Duplicates were removed using Covidence's automatic feature. Three independent researchers conducted the screening of the remaining articles. The screening process was conducted in two stages: first titles and abstracts of the relevant studies were reviewed and in the second stage the full texts of the selected studies were assessed for inclusion. Two researchers independently conducted the screening process, while a third researcher resolved all conflicts. During the final full-text screening, reasons for exclusion were documented. The screening process is depicted in Fig. 1 in accordance with the PRISMA-ScR statement [25].

#### Data extraction, analysis, and presentation

Data was extracted by one reviewer. For all included studies, data describing the study characteristics was extracted into Excel. Bibliographic data (including first author, publication year, publication format, and country of first author institution), study design, method, research objective, study population, study outcomes, and if applicable the intervention were extracted.

The extracted data are reported narratively. The study characteristics are depicted in a tabular form and summarized in text form. The following sections narratively describe information resources, information seeking behaviour, and information needs.

## Results

### Study selection and characteristics

The study selection process is depicted in Fig. 1. In total 2,942 articles were identified and imported into Covidence where 1,284 duplicates were removed automatically. The remaining 1,658 records underwent title and abstract screening. During this stage, 1,593 records were excluded, and the remaining 65 articles underwent full text screening. A total of 25 articles were included in the scoping review, of which 23 were written in English and two in German. The types of articles included one method paper, nine reviews, and 15 empirical studies, which were mostly cross-sectional. Geographically, studies were conducted in Europe (*n* = 7), Northern America (*n* = 7), Australia (*n* = 7), South America (*n* = 1), North Africa (*n* = 1), East Asia (*n* = 1), and Southeast Asia (*n* = 1). Most studies focused on information resources for endometriosis (*n* = 16). The second biggest category was information seeking behaviour (*n* = 7), including one study containing information on information seeking behaviour and information resources and one study describing information needs and information seeking behaviour. Lastly, four studies described the information needs of women with endometriosis. Table 2 depicts the study characteristics including the study objectives. The table does not show all extracted data. To enhance clarity and conciseness, Table 2 presents only the most crucial variables extracted during data analysis. For instance, the study methods have been summarised into the categories of quantitative, qualitative, and mixed methods for a more organised presentation.

**Table 2:** Study characteristics of included studies

Authors	Year	Country	Type of article	Study methods	Primary objective/research question
Abdulai et al	2022	Canada	Empirical study	Mixed methods	To evaluate the usability of a website for women with endometriosis-associated dyspareunia
Abdulai et al	2024	Canada	Review		To evaluate the quality, readability, and suitability of web-based resources on endometriosis-associated dyspareunia
Adler et al	2024	Australia	Review		To analyse the evidence-based nature of content on Instagram about endometriosis
Arena et al	2023	Italy	Empirical study	Quantitative	To assess how internet information-seeking affects endometriosis patients’ anxiety levels before and after outpatient medical examination
Bologna et al	2024	USA	Empirical study	Quantitative	To investigate aspects of endometriosis and aggregate user needs that emerge from two endometriosis online health communities
Carneiro et al	2020	Brazil	Empirical study	Quantitative	To assess the performance of a Facebook fan page developed by the Endometriosis Multidisciplinary Team of the Federal University of Minas Gerais
Chilton et al	2021	Australia	Empirical study	Quantitative	To assess what patients understand about endometriosis, where they obtain their information from, and to understand the perceptions of treatment options for endometriosis
Cox et al	2003	Australia	Empirical study	Mixed methods	To identify the information and support needs of women with endometriosis
Davidson	2005	USA	Review		To give an overview of endometriosis and to discusses selected endometriosis resources available on the Internet
Deevey	2005	USA	Review		To give an overview of information resources on endometriosis
Handelsman et al	2023	Australia	Empirical study	Mixed methods	To assess how endometriosis self-management strategies changed since the outbreak of COVID-19, and what the consequences of these changes are
Hirsch et al	2017	UK	Review		To assess World Wide Web pages providing women with endometriosis and the public information regarding the diagnosis and management of endometriosis
Holowka	2022	Canada	Empirical study	Mixed methods	To assess why and how people with endometriosis use social media
Knelangen et al	2010	Germany	Empirical study	Mixed methods	To understand the information needs of patients with Endometriosis and skin cancer
Lee et al	2022	Korea	Review		To analyse the content videos of endometriosis on YouTube
Li et al	2024	Australia	Method paper		to introduce a generic methodology designed to facilitate the development and implementation of health information recommendation features within web-based health applications
Moumane et al	2023	Morocco	Review		To study, analyse, and evaluate the functionalities and features of mobile apps that are specific to Endometriosis management and monitoring
Remes et al	2023	Finland	Empirical study	Qualitative	To describe endometriosis patients’ experiences of the counselling they need from the nurses through the digital care pathway
Sbaffi et al	2020	UK	Empirical study	Mixed methods	To assess and evaluate the role of the Internet in the diagnosis, treatment options and support of people living with endometriosis
Sirohi et al	2024	Australia	Review		To evaluate the quality and provide recommendations for high-quality endometriosis eHealth websites for the community and clinicians
Towne et al	2021	USA	Review		To analyse the nature and accuracy of social media (Facebook) content on endometriosis
van den Haspel et al	2022	Australia	Empirical study	Mixed methods	To analyse the use of social media for health in patients with endometriosis
Wilson et al	2020	Malaysia	Empirical study	Qualitative	To examine issues discussed in Malaysia on MyEndosis Facebook group
Zimmermann et al	2010	Germany	Empirical study	Quantitative	To identify information needs and knowledge gaps of women with endometriosis
Zugaj et al	2024	Germany	Empirical study	Qualitative	To investigate the influence of a healthcare app on the subjective experience of illness in patients with endometriosis

### Information resources for women with endometriosis

Studies on information resources for women with endometriosis can be divided into three categories: reviews analysing multiple resources, studies focusing on a singular resource, and one method paper demonstrating the application of a user-centred design approach. For the first category, nine reviews evaluated and compiled multiple information resources on endometriosis: five reviews focusing on websites and one review evaluating apps designed for women with endometriosis. Three reviews analysed social media platforms, such as Facebook, Instagram, and YouTube, regarding their content on endometriosis.

As mentioned, five reviews examined available online websites from formal and informal resources [29–33]. Davidson [29] focused on websites provided by government agencies, associations, and organisations to highlight unbiased, factual, and useful information for women with endometriosis. The article states a specific webpage and then gives a brief summary of the organisation, its services, and the information provided. In contrast, Deevey [30] presents online information resources from the perspective of a medical librarian. The article is structured by information sources such as search engines, medical sources or advocacy organisations, but the content overlaps substantially with the article by Davidson [29]. Hirsch et al. [31] took another approach and included the top results for search words on endometriosis from five different search engines. The overlap between websites mentioned in this study and the other two reviews is minimal. Sirohi et al. [33] evaluated 80 mainly Australian websites on endometriosis. The authors used the ENLIGHT quality assessment tool to evaluate the websites’ usability, visual design, user engagement, content, therapeutic persuasiveness, and therapeutic alliance. A review by Abdulai et al. [32] differed from the other reviews as it only focused on resources on endometriosis associated dyspareunia. The sixth review evaluated smartphone applications for women with endometriosis [34].

None of the six reviews provided a comprehensive discussion or detailed description of the information topics covered by the websites and smartphone applications. However, illustrative examples were provided.

One of the three reviews analysing social media platforms examined videos on endometriosis on YouTube [35]. The videos were grouped according to creators, those uploaded by the medical professionals and those by the nonmedical professionals. The medical group predominantly shared content focused on medical topics, including explanations and detailed surgical procedures, while the nonmedical group mostly uploaded videos about personal experiences. Another review analysing the nature of social media content focused on posts made on Facebook [36]. The study found most posts on Facebook regarding endometriosis focused on emotional support, rather than on information. The third review of social media content used a similar approach to Towne et al. [36] but evaluated Instagram posts [37].

Seven studies focused on individual information resources, such as websites, social media pages or apps. One evaluated the usability of a website for women with endometriosis-associated dyspareunia [38]. Another study presented a web-based platform for endometriosis patients called the EndoZone. [39] Only one study reviewed a mobile phone app offering information support for patients with endometriosis [40]. Four studies analysed social media pages as an information resource. One study focused on a Facebook page providing information, mostly on medical issues related to endometriosis and pelvic pain [41]. In contrast, Holowka et al. [21] used surveys and interviews to understand how and why women with endometriosis use social media in their patient journey. Wilson et al. [42] examined themes discussed by women with endometriosis in a Malaysian Facebook endometriosis group. Similarly, Bologna et al. [43] analysed the nature and content of posts made in endometriosis specific Reddit forums.

The topics covered by the information resources were either not or only partially described. Therefore, the following section cannot be seen as an extensive list of topics covered by information resources, but rather as an idea of what information resources can include.

The most common topic was treatment options for endometriosis, which was mentioned in nine studies [29, 35–37, 39–43]. This included alternative or complementary therapies [35–37, 40, 42], surgery [29, 35–37, 43], contraceptives [41, 43], and other medication [42, 43]. Side effects were another important issue regarding treatment options [41–43]. Endometriosis symptoms were frequently referenced [29, 30, 35–37, 42, 43]. One webpage exclusively focused on the symptom dyspareunia, which describes pain during intercourse. [38] The same first author published a review of information resources on dyspareunia [32]. Infertility [29, 36, 37, 41, 43] and in vitro fertilization (IVF) [41] were also an important foci. Research [29, 30, 33, 36, 37], lived experiences [30, 35, 42, 43], and diagnostic procedures [29, 35–37, 43] were similarly often mentioned.

Studies also included topics such as chat forums for informal advice [29, 30], endometriosis organisations and support groups [29, 33], information resources [29, 33, 37, 43], advocacy for endometriosis patients [36, 37], self-management strategies [29, 40, 42, 43], and how to communicate with healthcare providers [29, 42]. Other medical topics included deep infiltrating endometriosis [41], the cause of endometriosis [29, 30], endometriosis and cancer [41], medical terminology [29] as well as menstruation [29]. Nutrition was mentioned in three studies [37, 40, 43]. Two studies each provided information on scheduling appointments [41, 43], as well as insights into epidemiology and pathophysiology [36, 37]. Lastly, the following topics were mentioned once: information on how to deal with relationships as a woman with endometriosis [30], orthodox medicine and cures [37], and insurance policies and specialists [43].

### Information seeking behaviour

Studies including information seeking behaviour of women with endometriosis mostly focused on the internet as a resource, particularly social media. One study assessed how internet information-seeking affects women with endometriosis anxiety levels [44]. Two studies focused on the use of social media to acquire information about endometriosis [21, 45]. Another study examined the change in information seeking behaviour during the COVID-19 pandemic [46]. One study focused on the internet as an information resource and another focused on information seeking behaviour of women with endometriosis in general [47, 48]. Lastly, one study included a section in their survey on preferred ways to access information [49]. The preferred resources included advice from gynaecologists, general practitioners, and hospital staff, as well as printed and online materials.

Across all studies, the internet emerged as the most frequently utilised information resource, encompassing formal sources (e.g., patient organisations, medical societies) and informal sources (e.g., personal blogs).

Four studies did not explicitly describe the type of information sought by participants. One study identified preferred information sources; however, the article categorised the sought information as 'information needs,' which will be discussed in the subsequent section of this review [49]. In the two remaining studies, symptoms, and lived experiences were described as researched topics [21, 47]. Holowka et al. [21] further mentioned endometriosis as a general topic, diagnostics, and self-management strategies as areas of information interest.

### Information needs

Four studies analysed the information needs of women with endometriosis. To better understand the information needs of women with endometriosis, Zimmermann et al. [50] analysed 200 e-mails received by the German endometriosis association. Using a quantitative content analysis expressions of information needs were extracted. The results show women wanted information on the cause and epidemiology of endometriosis, as well as diagnostics, therapy, and infertility. Further women required information regarding self-help strategies and available health services. These findings were similar to the study by Knelangen et al. [51], in which a survey was used to assess the information needs of women with endometriosis as a foundation for the development of online health information. Using a Likert scale women could rate topics from “not interesting” to “very interesting”. All the following topics were rated as very interesting by most participants: cause, diagnostics, disease progression and consequences, treatment options including surgery, pharmaceuticals and alternative therapies, infertility, pain management as well as psychological and physical consequences of the disease. Via an open question, further information was requested on the topics of epidemiology, health services, and disease management.

The third study on information needs was conducted to understand which consultation topics should be covered by a digital care pathway nurse [52]. The themes were derived from qualitative interviews. The topics overlapped with the previously described findings but included additional categories. The overlapping themes were: cause, disease progression, epidemiology, treatment options including medication, surgery and alternative therapies, fertility, self-management and health services. Additional topics were comorbidities, endometriosis after menopause, treatment side effects, pregnancy, lifestyle factors, communication with social network and relationships, as well as additional information resources.

Lastly, Cox et al. [49] surveyed women with endometriosis regarding their information and support needs. The results align with the previously described information needs and particularly highlight the need for in-depth information about laparoscopic surgery and self-management.

## Discussion

This scoping review aimed to provide an overview of the literature on the format and scope of information resources on endometriosis as well as the information seeking behaviour and information needs of women with endometriosis. The findings indicate that the literature predominantly addresses information resources, with less attention given to information-seeking behaviour and information needs. The information seeking behaviour of women with endometriosis was mostly analysed as part of a broader research question and thus insufficiently explored. A gap exists in the English-language literature pertaining to information needs of women with endometriosis. Only two English articles were found describing the information needs of women with endometriosis. Overall, it can be stated that informational support for women with endometriosis is lacking in systematic evaluation.

The results further show that most information is given and sought through online channels such as websites and social media. A heavy focus on medical topics was evident. More holistic topics such as relationships and communication with healthcare providers and with their own social networks were of secondary importance. This prompts the question of whether the emphasis on medical topics primarily stems from the actual needs of patients or is driven by assumptions made by healthcare providers regarding patients' information requirements. As such, the lack of systematic and unbiased analysis is highlighted. The results of this scoping review show that evidence on the information seeking behaviour and the information needs of women with endometriosis is lacking.

### Further research

To provide information resources that fit the preferences of the target group, more baseline research needs to be conducted. This could be achieved by using a participatory research approach. Participatory research is an umbrella term for research methods, frameworks, and ideologies that include people with lived experience in research practices [53]. Rather than conducting research on a population, the focus shifts to conducting research with people with lived experience. To optimise the design of information resources, it is crucial to actively involve women with endometriosis throughout the entire process, from initial research to the final product, leveraging their valuable insights and unique lived experiences. The degree to which women with endometriosis could be included can vary from low-level consultation to shared decision-making and leadership [53].

No matter what approach is taken, it starts with assessing the information needs of women with endometriosis. It is thereby important to include as many subpopulations as possible. Information needs vary highly based on personal identity and context [26].

Although our understanding of the information needs of women with endometriosis is currently limited, insights from other medical conditions suggest that their information needs may vary across different stages of their journey [54–56]. Notably, these needs can evolve significantly from the time of diagnosis to several years later. This is further influenced by aging and thus the change in priorities and life goals [57]. Additionally, individual characteristics such as culture, occupation, and personality play a pivotal role in shaping the desire for information [57, 58]. It is essential to recognise the dynamic nature of these factors and their collective impact on the evolving information needs of women with endometriosis.

Secondly, once the information needs are assessed it is crucial to understand how women would like to access the information. This scoping review shows that most information was delivered through online sources, with a heavy focus on social media. Again, it is not evident whether this aligns with women’s preferences. Therefore, the information seeking behaviour of women with endometriosis should be more extensively researched. Studies should include personal as well as impersonal and digital and offline sources.

Based on these findings, information resources could be developed to align with the information needs and information seeking behaviour of women with endometriosis.

### Implications for practice and policy

Based on the current literature, the internet is the most common source of information on endometriosis. This includes websites as well as social media pages. This insight should be used to tailor relevant information specifically for women with endometriosis. Given the dynamic and evolving nature of media outlets, it is important to adapt to current trends. This could include short video content such as seen on TikTok or Instagram, using YouTube or podcasts for longer, more in-depth information. The possibilities of delivering information in the digitalisation age are highly varied; thus, it is important to assess the target group's preferences.

One of the biggest challenges, besides providing tailored information, is ensuring its accuracy, timeliness and objectivity. The study by Adler et al. [37] on informational posts on Instagram shows that scientific accuracy varied between topics. For example, information on surgical procedures was often not based on evidence. On the other hand, Towne et al. [36] found educational posts on Facebook to be accurate 93.93% of the time. Similarly, studies evaluating websites on endometriosis reported mixed results. The study by Hirsch et al. [31] concluded that no online websites provided high-quality, accurate, and credible information on endometriosis. However, it should be noted that this study was conducted in 2016 and since then the overall knowledge of endometriosis has advanced. Sirohi et al. [33] found four high-quality websites delivering information on endometriosis: Endometriosis Australia Facebook Page, Endometriosis UK, National Action Plan for Endometriosis on EndoActive, and Adenomyosis by the Medical Republic. The review of websites on dyspareunia by Abdulai et al. [32] reported equally high-quality websites but poor readability, thus making them unsuitable for the recommended reading level. The quality of information on endometriosis provided on social media and websites varies significantly, with some sources offering accurate, high-quality content, while others lack credibility and readability. The feasibility of online fact-checking effectively and expeditiously eradicating unfounded or biased information remains uncertain. In this context literacy and in particular, e-health literacy becomes vital. This spans across a wide variety of dimensions, including media literacy, which highlights the development of critical thinking skills [59]. Women need to be able to distinguish between reliable, evidence-based information and potentially biased or inaccurate content. This starts with evaluating the source and judging its credibility. It should be noted that this is not an easy task as even healthcare providers lack adequate education on endometriosis [17]. Determining the credibility of the source, specifically whether the information is provided by an individual with adequate expertise, can be challenging. Critical thinking becomes even more important and difficult when it comes to assessing medical research. As seen in this scoping review, research can be part of information resources. To understand research methodologies, results, and external validity a high level of scientific literacy is required [60].

A long-term solution needs to include better monitoring of online information, increased health literacy and more high-quality information resources. This will require cooperation between women with lived experience and women’s health organisations, legislators, and healthcare providers. In clinical practice, healthcare providers are encouraged to engage in open conversations with women with endometriosis to address their information needs. Providers can offer advice directly or refer women to reliable information sources. Additionally, healthcare professionals can invite women to share information they have independently found and discuss its empirical validity together.

### Limitations and strengths

This is the first scoping review to explore information resources on endometriosis as well as the information seeking behaviour and information needs of women with endometriosis. This therefore serves as a foundation for further exploration within this research domain. Nonetheless, it is important to acknowledge certain limitations in the methodology employed and the studies incorporated within the scoping review.

At the macro level of the scoping review, it cannot be guaranteed that all relevant studies have been identified. The term “information” can be used in a variety of contexts and was not considered suitable to be a MeSH Term. This decision was made in consultation with a librarian. Further, no grey literature was sought. Incorporating grey literature could have provided more extensive insights into the availability of informational support, particularly in relation to information resources. This scoping review aimed to understand what research had been undertaken and to identify existing research gaps. Thus, a grey literature search is more suitable for reviews focusing on evaluating available information, such as the ones by Deevey and Davidson [29, 30]. Although the screening process involved at least two reviewers, data extraction and analysis were carried out by a single reviewer. As a result, there is a potential risk of oversight or bias.

Following the JBI methodology for scoping reviews [24] no quality assessment of the included studies was conducted. Therefore, the quality of the studies was not assessed and as such any potential biases are unknown.

For the individual studies, it should be mentioned that in most articles the analysed themes were not described or discussed extensively. Therefore, the analysis of information topics covered cannot be seen as a comprehensive representation of either all information resources or information sought by women. This scoping review highlights the lack of systematic assessments in this space.

## Conclusion

This review highlights the lack of systematic assessments of endometriosis patients’ information needs and information seeking behaviour. While information sources exist, a bottom-up approach that includes women with endometriosis in the process is preferable to deliver relevant and tailored information. This could improve patient empowerment and support women in the long and difficult patient journey when living with endometriosis.

## Data Availability

No datasets were generated or analysed during the current study.

## Footnotes

1 This article will use the term women to refer to individuals assigned female at birth. The authors acknowledge trans, intersex, and non-binary people living with endometriosis.
